# Ultrahigh Stiffness and Energy Absorption Properties of Isotropic Metallic Closed Cell Microlattices

**DOI:** 10.1002/smll.73694

**Published:** 2026-05-07

**Authors:** Dominic Kang Jueh Lim, Chang Quan Lai

**Affiliations:** ^1^ School of Mechanical & Aerospace Engineering Nanyang Technological University Singapore Singapore; ^2^ Singapore Centre for 3D Printing School of Mechanical & Aerospace Engineering Nanyang Technological University Singapore Singapore; ^3^ School of Materials Science & Engineering Nanyang Technological University Singapore Singapore

**Keywords:** additive manufacturing, closed cell, energy absorption, metamaterials, microlattice, plate lattice

## Abstract

To validate leading theories on isotropic mechanical metamaterial designs, *pSC‐pFCC* closed cell microlattices are fabricated from SS304L sheets using the LAPIS additive manufacturing technique. By removing excess material at each layer, the fully enclosed voids in the lattice design are faithfully reproduced, confirmed by micro‐CT scan, without the need to introduce release holes for precursor materials. Material anisotropy caused by the layer‐by‐layer fabrication process is removed with a post‐print heat treatment. The microlattices exhibited highly similar elastic deformation in the <100> and <110> axes, with stiffnesses at the Hashin‐Shtrikman theoretical limit, as predicted previously. However, this isotropy in stress–strain response is unexpectedly extended to the plastic regime as well, even though the microlattices failed via plate buckling in <100> orientation, but by shear banding in <110>. Moreover, the microlattices also displayed remarkable specific energy absorption (15–33 J g^−1^) and energy absorption efficiencies up to 44%, at stresses as high as 410 MPa. Material work hardening is key to this breakthrough performance, as it raised the plateau stress of the plate buckling failure to approximately the same level as the stretch‐dominated elastic limit, which allowed ultrahigh stiffness to be united with excellent energy absorption characteristics in these mechanical metamaterials.

## Graphical abstract

True closed cell microlattices without holes are fabricated for the first time using a novel Laser Sheet Fusion additive manufacturing technique. These microlattices exhibited isotropy in stiffness and surprisingly, strength and plasticity as well, despite orientation‐dependent failure mechanisms. Furthermore, material work hardening is shown to be key in achieving high stiffness and excellent cushioning properties simultaneously in lattices.

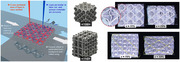

## 1 Introduction

Advancements in lightweight structural materials remain key to sectors such as automobiles, aviation and satellite engineering where reductions in structural weight can lead to significant cost savings from improved fuel efficiencies and decreased carbon emissions. For example, a 18% reduction in airframe weight has been estimated to yield cost savings of $16 million USD over an aircraft's operational lifetime [1], while every kilogram shaved off a satellite can reduce launch costs by $2000 – $20 000 USD [2]. Similarly, a reduction of automobile weight by 10% can improve fuel efficiency by 6% – 8% [3], and a decrease of carbon dioxide emissions from 300 to 150 g km^−1^ can lighten the cost of car ownership by ∼ $23 000 USD in Singapore, as a result of sustainability‐driven subsidies [4].

One promising strategy for producing lightweight structural materials involves the use of lattice metamaterials with architected geometries optimized for mechanical performance [5]. However, like naturally occurring atomic crystal structures, these lattices are often anisotropic, exhibiting direction‐dependent stiffness and strength [6], which can be undesirable for engineering applications facing multiaxial loads. Moreover, anisotropic materials (up to 21 independent elastic constants) [7] often require more time and computational resources to simulate their mechanical response compared to isotropic materials (2 independent elastic constants), making the latter the preferred choice for engineering designs.

Although the theoretical limit for the elastic modulus of isotropic lattices was derived by Hashin and Shtrikman in 1963 [8], actual designs of isotropic lattices with periodic unit cells were not introduced until 1992, in a publication by Lake and Klang [9]. More recently, Gurtner and Durand presented an isotropic design based on the faces of the Kelvin cell geometry [10] while Xu et al. showed that isotropy can be achieved by tuning truss diameter ratios in *FCC‐BCC* (Face Centered Cubic – Body Centered Cubic) and diamond‐cubic structures [11]. Subsequently, a ternary design map indicating the different fractions of *SC* (Simple Cubic), *BCC*, and *FCC* trusses that can be hybridized into isotropic lattices was reported by two independent groups [6, 12]. However, truss lattices are structurally inefficient, and thus, the isotropic variants typically achieve stiffness values less than one‐third that of the Hashin‐Shtrikman (H‐S) upper bound. Nevertheless, they have been more intensively studied to date, possibly because they are easier to fabricate for experimental validation using techniques such as additive manufacturing, investment casting [13] and brazing [14].

To obtain isotropic elastic modulus close to the H‐S upper bound, researchers have recently turned to closed cell plate lattices. Berger et al. first showed that replacing beam elements in isotropic truss lattices with plates that are oriented along planes of highest nodal density can lead to a hybrid *pSC‐pFCC* (plate Simple Cubic—plate Face Centered Cubic) structure with an isotropic elastic modulus that approaches the H‐S upper bound [15]. Expanding on this finding, Tancogne‐Dejean et al. went further and demonstrated that high isotropic elastic modulus can be achieved by combining *pSC*, *pFCC*, and *pBCC* (plate Body Centered Cubic) structures in a volume ratio of 0.4 – 0.25*x*:0.6 – 0.75*x*:*x*, where 0 ≤ *x* ≤ 0.8 [16]. These proposed structures, however, were based on analyses that assumed plane stress condition (i.e. infinitesimally thin plates) and thus, at higher relative densities, the simulated moduli of the structures fell short of the H‐S upper bound. To overcome this limitation, Feng et al. introduced a numerically‐derived geometry that consists of *pFCC* structures nested within the cavities of a larger *pSC* geometry, bringing the modulus to within 2.1% from the H‐S upper bound at a relative density of 0.6 [17], the closest approach to date.

In addition to enhanced mechanical performance, plate lattices have demonstrated improved capabilities in sound absorption [18, 19], thermal insulation [20, 21], and vibration damping [22, 23]. Despite advances in the design of isotropic plate lattice, the benefits of these closed cell metamaterials have yet to be realized practically due to manufacturing limitations. Currently, true closed cell manufacturing of isotropic materials can only produce solid foams with random placement of voids, which deviate from the deterministic geometries of plate lattices [24]. Additive manufacturing, on the other hand, is incompatible with closed cell designs as its layer‐by‐layer fabrication process can cause precursor materials, such as unfused powder or uncured resin, to be trapped and isolated in the cavities of the geometry. To mitigate this issue, researchers have introduced holes in the designs to facilitate precursor material removal [16, 25, 26, 27], but this effectively transforms the design into an open cell structure, compromising its stiffness, strength and energy absorption properties [26, 28]. Furthermore, this method is unlikely to work well in structures with a large number of unit cells, as the geometrical tortuosity of the lattices makes it difficult to fully remove material trapped in the innermost cells, which could lead to higher‐than‐expected relative densities and consequently, lower specific stiffness and strength [17].

In addition, the reports to date generally do not i) conduct cross‐sectioning or micro‐computed tomography (micro‐CT) to confirm that the enclosed spaces in the pseudo‐closed cell lattices were truly vacant, or ii) perform mechanical testing to verify isotropy in both the build and transverse axis [16, 17, 25, 29, 30, 31]. As a result, studies on true closed cell plate lattices without holes have thus far been realized only with fused deposition modeling (FDM) [32] and sheet lamination techniques [33], which permit overhang formation without trapping precursor material. However, these processes also introduce permanent anisotropy to the printed structures due to imperfections in bonding between the layers, which defeats the objective of obtaining isotropic closed cell structures.

To overcome these manufacturing limitations, we employed the LAPIS technique, a custom laser sheet fusion additive manufacturing process [34] to fabricate true closed cell microlattices using SS304L metal sheets. The excess material for each layer was removed immediately after patterning, rather than at the end of the additive manufacturing process, thereby ensuring that no material will be trapped in the enclosed spaces of the closed cell design. Post‐print annealing of the microlattices was employed to recrystallize the metal grains and eliminate material anisotropies introduced during the printing. The *pSC‐pFCC* (*a.k.a*. Cubic Octet) design was selected for this study as it has been widely studied relative to other closed cell designs. The elastoplastic mechanical response of *pSC‐pFCC* under uniaxial compression was investigated using finite element simulations and validated experimentally in the <100> and <110> orientations. Their deformation process and energy absorption performance were characterized in detail for the first time and compared against similar mechanical metamaterials in the literature.

## 2 Methods

### 2.1 Fabrication of True Closed Cell Microlattices

A laser sheet fusion additive manufacturing technique, Laser Pulsed Integration of Sheets (LAPIS) [34], was employed to fabricate plate microlattices with fully enclosed air spaces. No holes were introduced to the design to facilitate precursor removal. The microlattices were constructed from 0.05 mm thick stainless steel 304L sheets (i.e. Build axis resolution = 0.05 mm).

**FIGURE 1:**
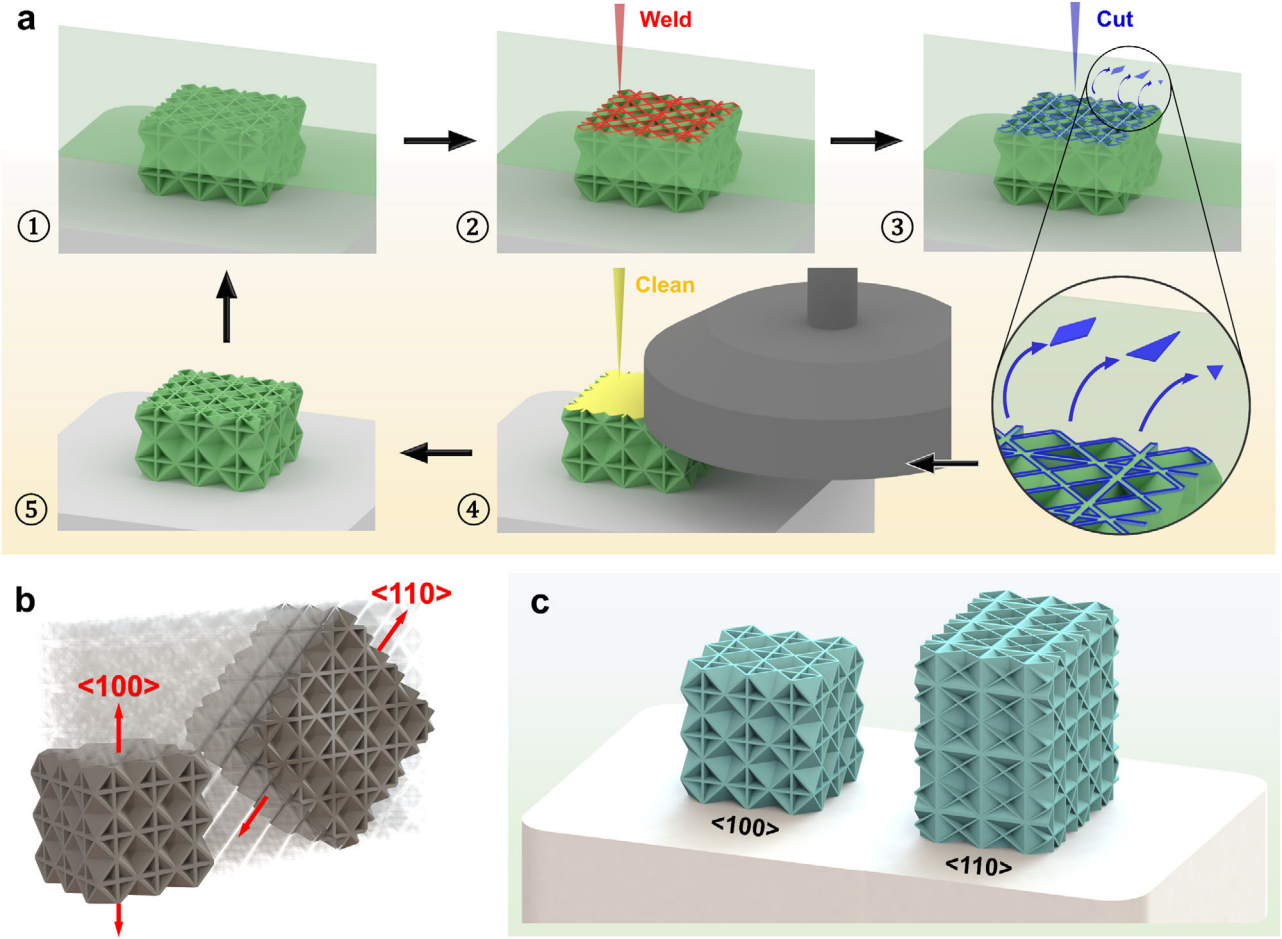
(a) Schematic illustration of the LAPIS additive manufacturing process used to fabricate the *pSC‐pFCC* closed cell microlattices without the need to remove precursor materials through release holes. (b) *pSC‐pFCC* microlattice oriented in the <100> and <110> directions. (c) Schematic illustrations of additively manufactured <100> and <110> *pSC‐pFCC* microlattices.

The process began with the introduction of a fresh metal sheet on top of the build (Figure 1a ①). Laser welding was performed using a 1064 nm nanosecond pulsed laser (Figure 1a ②), followed by laser cutting of the design contours (Figure 1a③). Excess material was removed from the print via suction at every layer, to avoid material entrapment. Next, a laser cleaning step was applied to remove surface oxides [34]. Finally, a deburring step was performed to remove any surface asperities (Figure 1a ④). These steps were repeated until the microlattice design was fully realized (Figure 1a ⑤). All processes were carried out under ambient conditions.

The *pSC‐pFCC* isotropic lattice geometry (Figure 1b,c) is composed of the plate SC design, with a plate member thickness of *t*_SC_, and a plate FCC design, with a plate member thickness of *t*_FCC_. The ratio of the plate thicknesses had previously been established as [15]:

(1) $$\;\frac{t_{SC}}{t_{FCC}}=\frac{8\sqrt{3}}{9}$$

Due to the Build axis resolution (Z: 0.05 mm) and limited fabrication height (Z‐axis: 15 mm) of our current LAPIS setup, the thicknesses of the SC plate members were selected as 0.15, 0.20, 0.25, and 0.30 mm, which correspond to relative densities of *ρ/ρ_s_* = 0.29, 0.37, 0.44, and 0.51 respectively (Figure S1). Note that *t*_SC_ had to be commensurate with the sheet thickness of 0.05 mm, to ensure the build in the Z‐axis required an integer number of sheets. The fabricated microlattices consisted of 3 × 3 × 3 unit cells to minimize size effects (Figure S2) and had overall dimensions of 10.5 mm × 10.5 mm × 10.5 mm for the <100> orientation, and 14.85 mm × 14.85 mm × 10.5 mm for the <110> orientation (Figure 1b,c). A total of twelve microlattice samples were fabricated in this study. For each of the four relative densities, three configurations were produced and tested: <100> orientation loaded along the build direction, <100> orientation loaded along the transverse direction, and <110> orientation loaded along the build direction.

### 2.2 Micro‐Computed Tomography

Micro‐Computed Tomography (Micro‐CT) scan was performed using a Zeiss XRadia Versa 620 to assess the internal structure and verify the absence of trapped material within the closed cell microlattices. The system was equipped with a 0.4× objective lens, and the sample was scanned with a voltage of 160 kV and power setting of 25 W, with an exposure time of 1s per projection. An isotropic voxel size of 15.5 µm was used, and a total of 801 X‐ray projections were acquired to reconstruct the 3D structure of the microlattice using Dragonfly 3D World ZEISS software.

### 2.3 Heat Treatment

Annealing of the microlattices was performed using a Camco G‐VAC‐20TL vacuum furnace under a high vacuum environment (< 10^−6^ Torr). Samples were heated at a controlled ramp rate of 10°C min^−1^ to a peak temperature of 1200°C, held isothermally for 2 h, and subsequently cooled to room temperature at a controlled rate of 3.33°C min^−1^.

### 2.4 Electron Backscatter Diffraction (EBSD)

To characterize the effects of the heat treatment process on the resulting microstructure, cube samples were sectioned using a diamond saw and mounted in conductive epoxy. The cross‐sectional surfaces were sequentially polished using FEPA grit sizes of P320, P800, P1200, and P2000, followed by 3 and 1 µm diamond suspensions (Struers LaboPol‐20). A final chemical mechanical polishing step was performed using a 0.04 µm colloidal silica suspension (OP‐S) to prepare the surface for EBSD characterization.

Microstructural mapping was performed using a field emission scanning electron microscope (JEOL JSM‐7800F PRIME) equipped with an Oxford Symmetry EBSD detector. The scans were conducted with an accelerating voltage of 20 kV, a beam current of 20 nA, and step size of 0.5 µm. EBSD data acquisition and analysis were carried out using Oxford Aztec and AztecCrystal software respectively. The raw EBSD data was refined by removing zero‐solution points using a neighbor extrapolation approach (up to five neighboring pixels) to eliminate stray unindexed points. High angle grain boundaries were identified by a misorientation angle exceeding 15 degrees.

### 2.5 Quasistatic Compression Test

Uniaxial compression tests were performed using a Shimadzu Autograph AGS‐X machine equipped with a 50 kN load cell, using a quasi‐static strain rate of 0.001 s^−1^. For strain measurements in the elastic regime, the vertical distance between the top and bottom surfaces of the microlattices was extracted using the open‐source Tracker software [35]. For strain measurements in the plastic deformation regime (strain, *ε* > 1%), the platen displacement was tracked using a video extensometer.

### 2.6 Finite Element Analysis

Finite element simulations were conducted using ABAQUS Explicit. The CAD geometries were meshed using second‐order tetrahedron C3D10 elements, with mesh sizes at least one‐third of the plate thickness of microlattices to ensure good mesh convergence (Figure S3). A custom elastic–plastic material model was assigned based on experimentally obtained Young's modulus and true stress–strain tensile data of an annealed SS304L dogbone‐shaped sample (Figure S4). To simulate the quasistatic compressive response of the closed cell microlattice structures, a rigid plate was positioned atop the microlattice and displaced downwards. General contact conditions were defined with a friction coefficient of 0.3 [36]. The reaction force and displacement of the top plate were then extracted to compute the simulated stress–strain curves of the microlattices.

Symmetry boundary conditions were applied to reduce computational time, i.e. microlattices were constrained with ZSYMM (UR1 = UR2 = U3 = 0) on the horizontal XY‐plane. For the <110> microlattices, an additional XSYMM constraint (U1 = UR2 = UR3 = 0) was applied on the YZ‐plane, corresponding to the (110)‐plane of the *pSC‐pFCC* unit cell. Although symmetry also exists in the XZ‐plane, the plane bisects a vertical plate member and would artificially constrain out‐of‐plane buckling if applied. Therefore, symmetry conditions were not imposed along this plane. In addition, simulations on <111> microlattices had also been conducted, but because the structures are too tall to be fabricated by our present setup, the simulation‐only results and related discussion are included in Figure S5, rather than the main text.

## 3 Results

### 3.1 Effects of Heat Treatment

**FIGURE 2:**
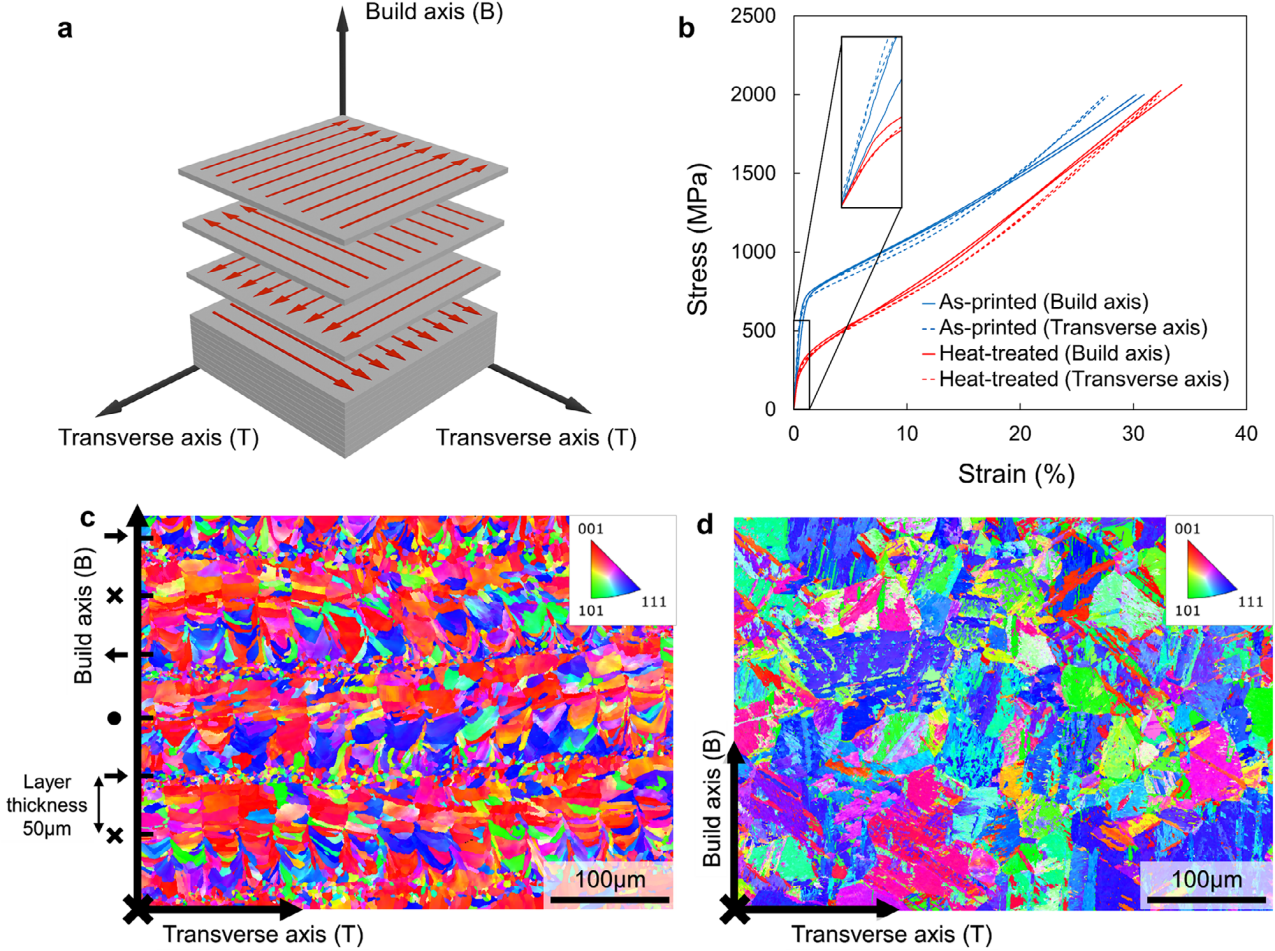
(a) Schematic diagram illustrating the fabrication strategy of rotating laser scans 90° between successive layers. (b) Compressive stress‐strain response of as‐printed (AP) and heat‐treated (HT) cubes tested along both the Build (B) and Transverse (T) axes. (c) Electron backscatter diffraction (EBSD) inverse pole figure (IPF) maps of as‐printed samples (side view), showing grain alignment and crystallographic texturing. Laser scan directions for each layer (50 µm thick) are indicated in the vertical axis. (d) IPF map of heat treated samples exhibiting grain growth and homogenization after annealing.

5 mm × 5 mm × 5 mm cubes were fabricated with the LAPIS system. Welding was carried out unidirectionally for each layer, and rotated 90° with each new successive layer, so the cube was indistinguishable in the in‐plane axes (i.e. XY) (Figure 2a). Compression tests were conducted on as‐printed (AP) cubes along the build (AP‐B) axis, as well as the transverse (AP‐T) axis, with two samples tested per orientation. AP‐B and AP‐T samples exhibited only a minor difference in yield strength, measured at 675 ± 7 and 630 ± 28 MPa, respectively. However, their elastic moduli were significantly different – 97 ± 13 GPa for AP‐B and 122 ± 6 GPa for AP‐T (Figure 2b, blue lines). EBSD analysis of the AP cubes revealed a pronounced crystallographic texture along the transverse axes (Figure 2c), induced by the directional laser processing, leading to anisotropy in the printed material. This texture‐driven anisotropy is also commonly reported in PBF processes, where directional solidification under thermal gradients results in preferential crystallographic orientations and anisotropic mechanical behavior [37, 38].

To realize the isotropic *pSC‐pFCC* geometry, however, the constitutive material is required to be isotropic [15, 16]. Therefore, annealing of the samples at 1200°C was carried out for 2 h to induce recrystallization and promote grain uniformity in the printed metallic material. The effectiveness of the treatment was confirmed by inverse pole figure (IPF) maps of heat‐treated (HT) samples, which showed a random distribution of grains and crystallographic textures (Figure 2d). Compression tests on HT‐B and HT‐T samples revealed virtually identical mechanical responses in both elastic and plastic regimes (Figure 2b, red lines) – the elastic modulus was 64.5 ± 5 GPa for HT‐B and 65.5 ± 2 GPa for HT‐T, while the yield strengths were 257.5 ± 25 and 255 ± 7 MPa respectively. These results indicate that the heat treatment successfully restored isotropy in the constitutive SS304L material. The fall in the yield strength from ≈650 to ≈250 MPa can be primarily attributed to the enlargement of grains after heat treatment (i.e. Hall ‐Petch effect), from an area‐weighted mean of 14.17 µm ± 4.58 µm to 24.75 µm ± 5.40 µm (Figure 2d).

### 3.2 Additively Manufactured Closed Cell Microlattices

**FIGURE 3:**
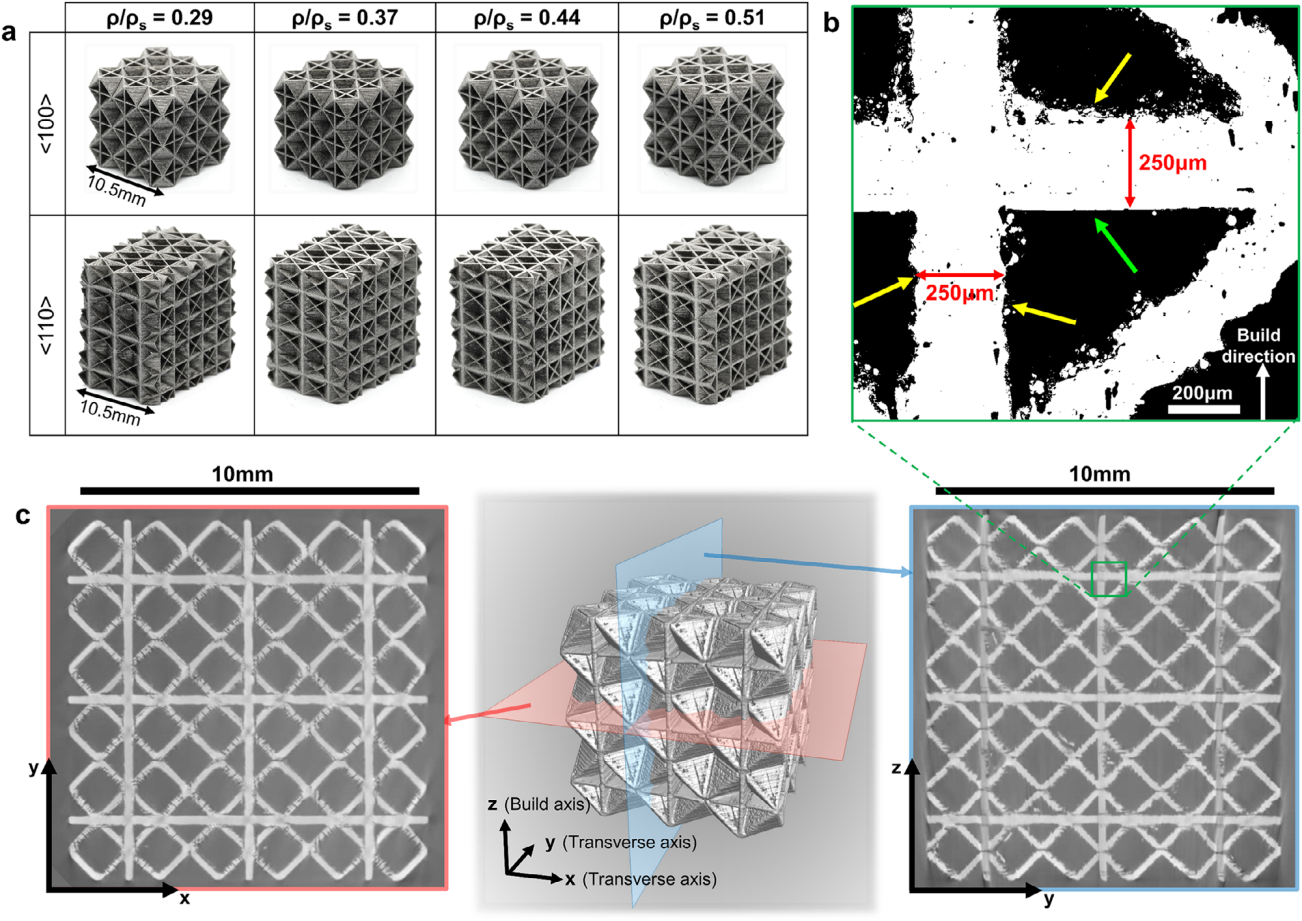
(a) Additively manufactured <100> and <110> *pSC‐pFCC* closed cell microlattices with relative densities, *ρ/ρ_s_* = 0.29, 0.37, 0.44, and 0.51. (b) Cross‐sectioned optical microscopy of <100> *pSC‐pFCC* closed cell microlattice with *ρ/ρ_s_* = 0.44 and SC plate thickness, *t_sc_* = 250 µm. (c) Micro‐CT scan of <100> *pSC‐pFCC* closed cell microlattice with *ρ/ρ_s_* = 0.37. Cross‐sectional images in the transverse‐transverse plane (red plane, left) and build‐transverse plane (blue plane, right).

*pSC‐pFCC* closed cell microlattices with four different relative densities (*ρ/ρ_s_* = 0.29, 0.37, 0.44 and 0.51) were fabricated in crystallographic orientations of <100> and <110> (Figure 3a). To evaluate the dimensional fidelity of the prints, a representative microlattice with *ρ/ρ_s_* = 0.44 was cross‐sectioned and examined with optical microscopy (Figure 3b). The SC plate thickness was measured to be 250 µm, consistent with the intended design. However, defects which distorted the dimensions of the prints at various locations could also be observed. Specifically, the top surface and sides of the SC plates (Figure 3b, yellow arrows), as well as sidewalls of the diagonal FCC plates, exhibited rougher surfaces and local thickness variations, as they had been subjected to laser welding and/ o cutting. The underside of the SC plate (green arrow in Figure 3b), however, was virtually free of defects since it did not undergo laser processing, allowing it to retain its original flatness from the precursor sheet.

Going further, a micro‐CT scan was also performed to investigate the presence of trapped materials within the closed cell structures. Even though no holes had been introduced to the lattice design to facilitate the removal of precursor material, it is clear that there was no residue left in the isolated air spaces within the *pSC‐pFCC* closed cell structure. Furthermore, comparing the cross‐sections in the transverse‐transverse plane (*i.e*. XY plane) and build‐transverse plane (*i.e*. YZ plane) (Figure 3c), it can be observed that the SC plates were straighter in the transverse‐transverse plane (red) than the build‐transverse plane (blue). This is because the geometrical pattern of *pSC‐pFCC* in the XY transverse‐transverse plane was realized in a single laser sequence for each layer, while the vertical SC plates in the build‐transverse plane was constructed sequentially, as multiple laser sequences built up the geometry layer‐by‐layer. Therefore, slight differences in the patterns between layers, due to imperfections such as those shown in Figure 3b, can give rise to minute eccentricities in SC plates in the build direction.

### 3.3 Mechanical Response

#### 3.3.1 Build Axis vs Transverse Axis

**FIGURE 4:**
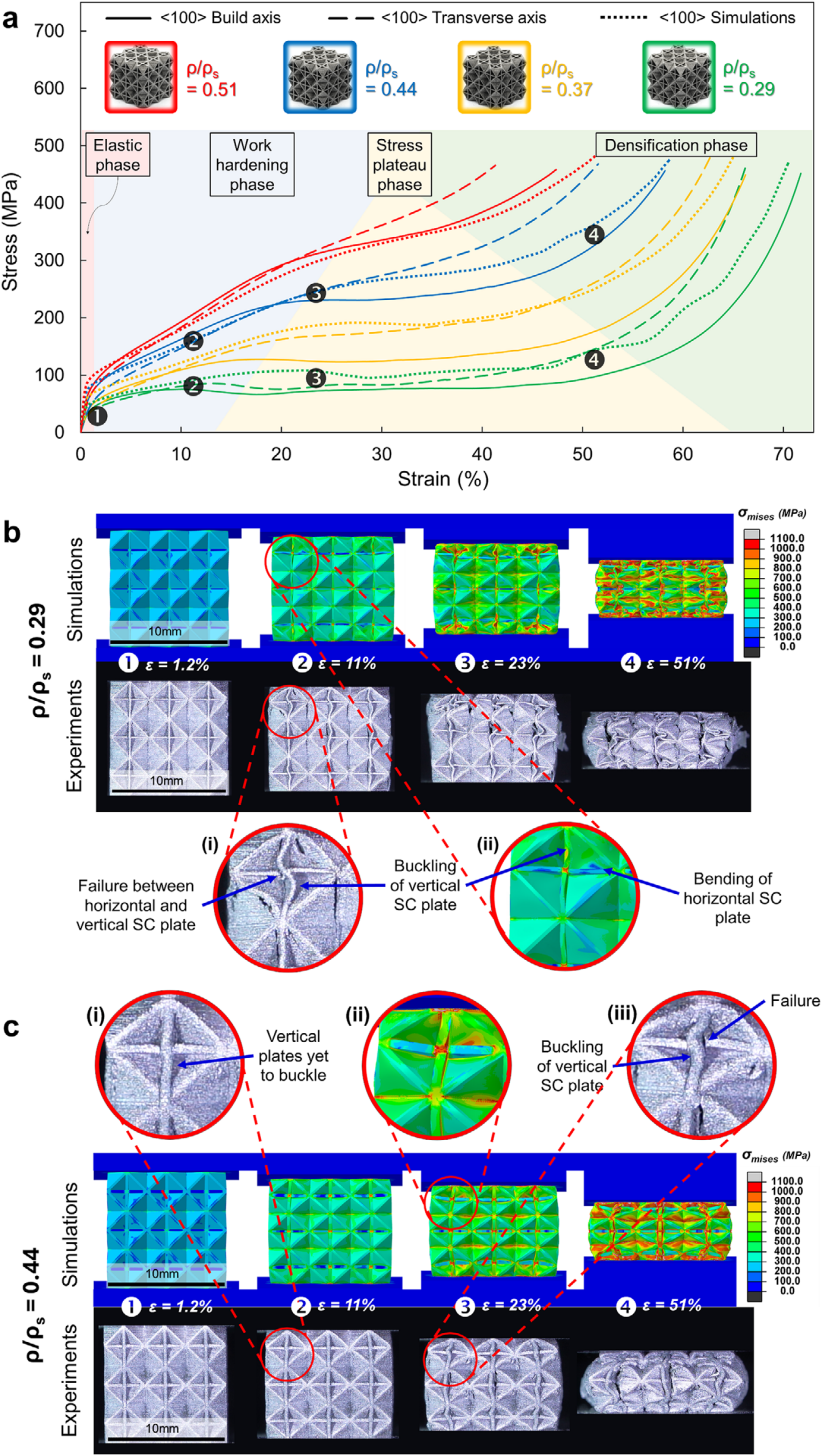
(a) Experimental and simulated (dotted lines) compressive stress–strain curves of <100>‐oriented *pSC‐pFCC* closed cell microlattices loaded in the Build (solid lines) and Transverse (dashed lines) axes, for *ρ/ρ_s_* = 0.29, 0.37, 0.44, and 0.51. (b) Experimental and simulated mechanical responses of *pSC‐pFCC* closed cell microlattices under uniaxial compression along the Build axis, for *ρ/ρ_s_* = 0.29 and (b) *ρ/ρ_s_* = 0.44. (Insets) Magnified views of the experimental and simulated microlattices, showing premature plate failure, buckling of plates, and regions of high stress concentration.

The <100> *pSC‐pFCC* microlattices were subjected to compression testing in both the build and transverse axes to evaluate their isotropy (Figure 4a). Their elastoplastic stress‐strain response can be divided into four distinct regimes to facilitate discussion.

Stage I — An initial linear stress‐strain region where the microlattices deform elastically and reversibly. Here, the measured elastic modulus in both axes differed by less than 3% across all relative densities, indicating that the microlattices were elastically similar in the *X*, *Y* and *Z* axes. This implies excellent layer bonding in the printed microlattices and indicates that the LAPIS additive manufacturing process, together with the post‐print heat treatment, did not impart anisotropy to the printed microlattices.

Stage II — Past the yield point, the microlattice deformation entered into a work hardening phase. During this time, the deformation was plastic (permanent) and the SS304L material at high stress regions began to strain harden, leading to a continued rise in stress sustained by the microlattices. To confirm this, finite element (FE) simulations of the microlattice compression, which incorporated work hardening data, were carried out (Figure S4) and shown to agree closely with the experimental results. The microlattices continue to exhibit similar mechanical response in both the Transverse and Build axes during this phase.

Stage III — The stress plateau regime, where the stresses were observed to level off. The onset of this regime corresponded to buckling and fracturing of plate elements in the microlattices, which had a countereffect to the ongoing strain hardening. The net result was that the load‐bearing ability of the structure remained approximately constant in this regime, with little to no undulations in the stress that is indicative of a layer‐by‐layer fracture sequence [39, 40], even for the lowest relative density of 0.29. However, unlike Stages I and II, the plateau stress in the Build axis tends to be lower than in the Transverse axis (e.g. ≈25% (40 MPa) lower for *ρ/ρ_s_* = 0.37), indicating a breakdown of geometrical isotropy. This result can be attributed to the imperfections in the vertical plates in the Build axis, discussed previously in Figure 3b, which rendered them more susceptible to premature buckling and collapse. This reasoning is further supported by the FE results, which did not account for manufacturing imperfections and thus, more closely matched the stress–strain response of the defect‐free microlattices in the Transverse axis, particularly at lower relative densities.

Stage IV — Finally, at much higher strains, the microlattice deformations entered the densification regime, during which the plate elements came into contact and deformed collectively. At this point, the topology and mechanical response of the material had changed, resembling more closely to that of a bulk material. For the microlattices with higher relative densities, the transition to the densification regime occurred shortly after entering the stress plateau regime, whereas microlattices with lower relative density exhibited an extended stress plateau regime. This is expected as the thicker plate members in high relative density microlattices can come into contact more easily, at lower compressive strains.

#### 3.3.2 Deformation Mechanisms in the <100> Orientation

A closer examination of the simulated and actual deformation of the *pSC‐pFCC* closed cell microlattices shows that the structures experienced a relatively uniform stress distribution in both the elastic (Stage I) and work hardening regimes (Stage II) (Figure 4b,c). This suggests that the plate members underwent stretch‐/ compression‐ dominated deformation in these stages, as bending and buckling deformation tend to result in uneven stresses and strains in a structure.

For the low relative density microlattices (*ρ/ρ_s_* = 0.29), severe plate buckling began to set in at 11% strain (*i.e. ε* ≈11%) (Figure 4b(i)). Because the vertical plates intersect with the horizontal plates to form a central node in each *pSC‐pFCC* unit cell, the vertical plates were constrained and could not deflect at the node. This led them to adopt a second harmonic buckling shape (Figure 4b(i)) and the subsequent rotation of the node (Figure 4b(ii)), in turn, caused the horizontal plates to shear and fracture in the experiments (Figure 4b(i)). The subsequent loss of constraint on node rotation provided by these horizontal plates then caused the buckling shape of the vertical plates to become more pronounced. The simulations, however, did not capture the fracture of the horizontal plates at *ε* ≈11%, as the models lacked the imperfections of the printed microlattices which likely triggered the early commencement of plate buckling. As a result, the simulations overestimated the load‐bearing capability of the microlattices and consistently predicted a delayed onset of the stress plateau regime (Stage III) (Figure 4a).

At higher relative densities (*ρ/ρ_s_* = 0.44), the vertical plates in *pSC‐pFCC* microlattices exhibited little deflection at 11% strain (Figure 4c(i)), and buckling was not observed until the strain reached 23% (Figure 4c(ii)). This higher failure strain was key to the delayed transition into Stage III deformation for the microlattice with *ρ/ρ_s_* = 0.44, as compared to the microlattice with *ρ/ρ_s_* = 0.29, and can be attributed to the lower aspect ratio (i.e. length/ thickness) of the plate members. Only one of the two horizontal plates fractured and detached from the central node (Figure 4c(iii)), possibly because the surface defects observed in Figure 3b did not affect the thicker plates as much as the thinner plates in the microlattice with *ρ/ρ_s_* = 0.29. As a result, the simulations were able to reflect the actual stress‐strain response of the *pSC‐pFCC* closed cell microlattices at higher relative densities more accurately (Figure 4a).

#### 3.3.3 <100> vs <110> Lattice Orientations

**FIGURE 5:**
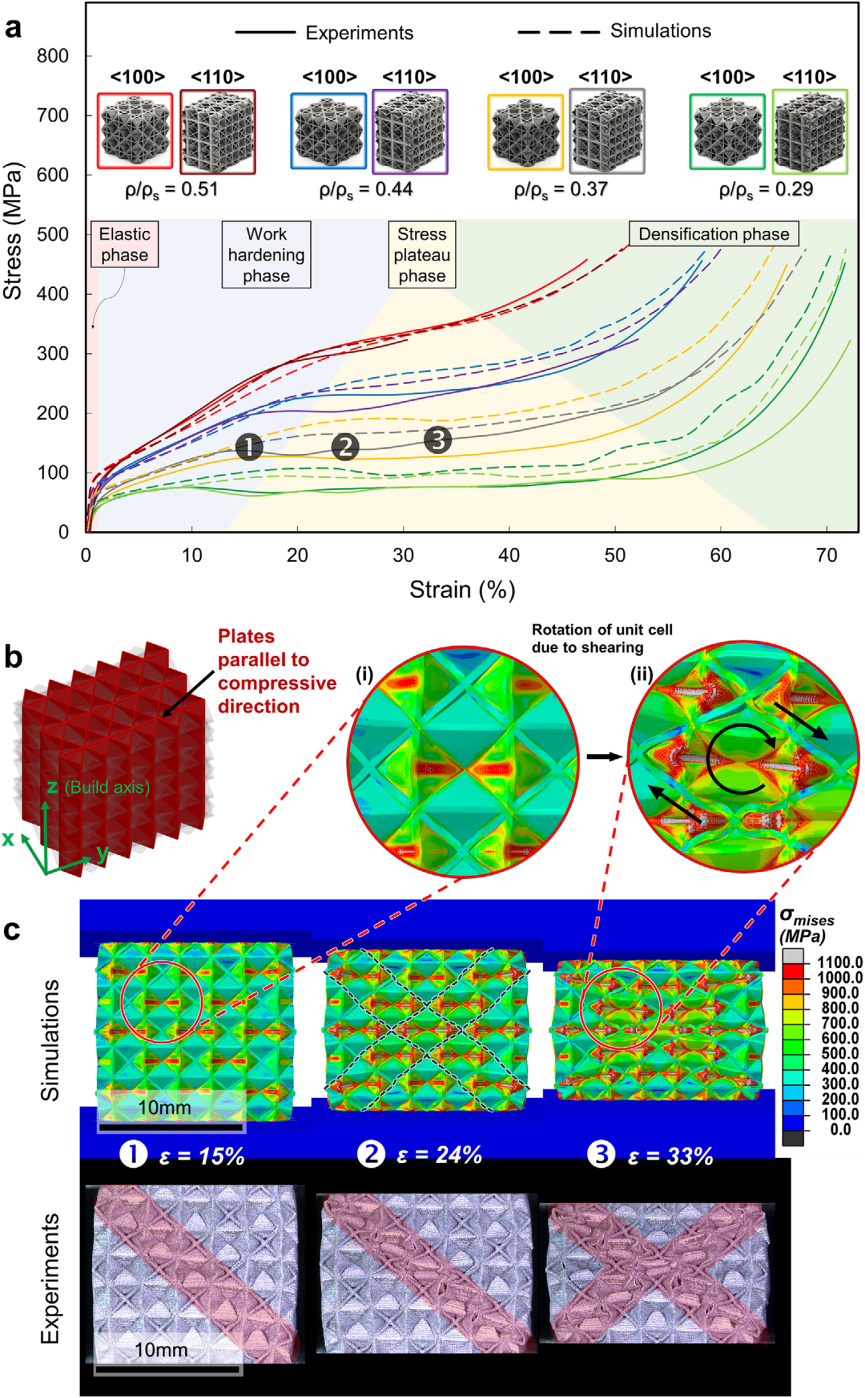
(a) Compressive stress–strain curves of *pSC‐pFCC* closed cell microlattices with <100> and <110> orientations for different relative densities in the Build axis. (b) Schematic illustration of the <110>‐oriented microlattice. Plate members parallel to the <110> loading direction are highlighted in red. (c) Experimental and simulated uniaxial compressive response of <110>‐oriented microlattice (*ρ/ρ_s_* = 0.37). Insets show magnified views of the simulated microlattices. Black arrows indicate the directions of the shear stresses and the resultant rotation of unit cell. Red overlays have been added to the experimental microlattices to highlight the macroscopic shear bands.

To ascertain that the isotropy of the *pSC‐pFCC* closed cell microlattices went beyond the <100> axes, their mechanical response in the <110> axis was also characterized and found to match closely with the <100> curves across the 4 distinct phases of deformation, in both simulations and experiments (Figure 5a). For instance, the elastic moduli and yield stresses of the <100> and <110> microlattices did not deviate by more than 17% and 10% from each other, and the experimental stress‐strain curves (solid lines) for both orientations were nearly identical in the work hardening (Stage II) and stress plateau regime (Stage III).

While the isotropy in the elastic regime was previously predicted from first principles calculations, its extension to the plastic regime was unexpected. One possible reason could be the presence of vertical plate members (isolated and highlighted in red in Figure 5b) in the <110> *pSC‐pFCC* geometry, which is similar in number and size to the vertical plate members present in the <100> orientation. However, unlike the <100> microlattices, where the vertical plates were contributed by two of the three plates of the SC sublattice, vertical plates for the <110> microlattice consisted of members from both the SC and FCC substructures. Furthermore, the vertical plates were arranged in a triangular tessellation (Figure 5b), which helped constrain in‐plane bending and rotation of the plates, rendering the structure more rigid and resistant to buckling compared to the square‐tessellated aligned plates in the <100> microlattices.

As a result, buckling was not observed to be the main deformation mode for the <110> microlattices. Instead, the <110> microlattices failed when shear bands first appeared at a 45° angle, along one of the diagonals (red overlays in experimental images in Figure 5c), before they intensified and developed in the opposite diagonal as well. The shear stresses were observed to buckle the diagonally‐oriented plates (black dotted lines in simulation image for Figure 5c, *ε* = 24%), and also distort and rotate the unit cells (Figure 5c, insets). According to the simulations, the shear band development was expected to cause the deformation to transition to Stage III at *ε* = 24%, but manufacturing imperfections caused the failure to initiate earlier, at *ε* = 15% (Figure 5c). Nevertheless, it is remarkable how different failure mechanisms in the <100> (buckling) and <110> (shearing) orientations led to the same effective stress–strain response.

### 3.4 Mechanical Properties

#### 3.4.1 Stiffness and Strength

**FIGURE 6:**
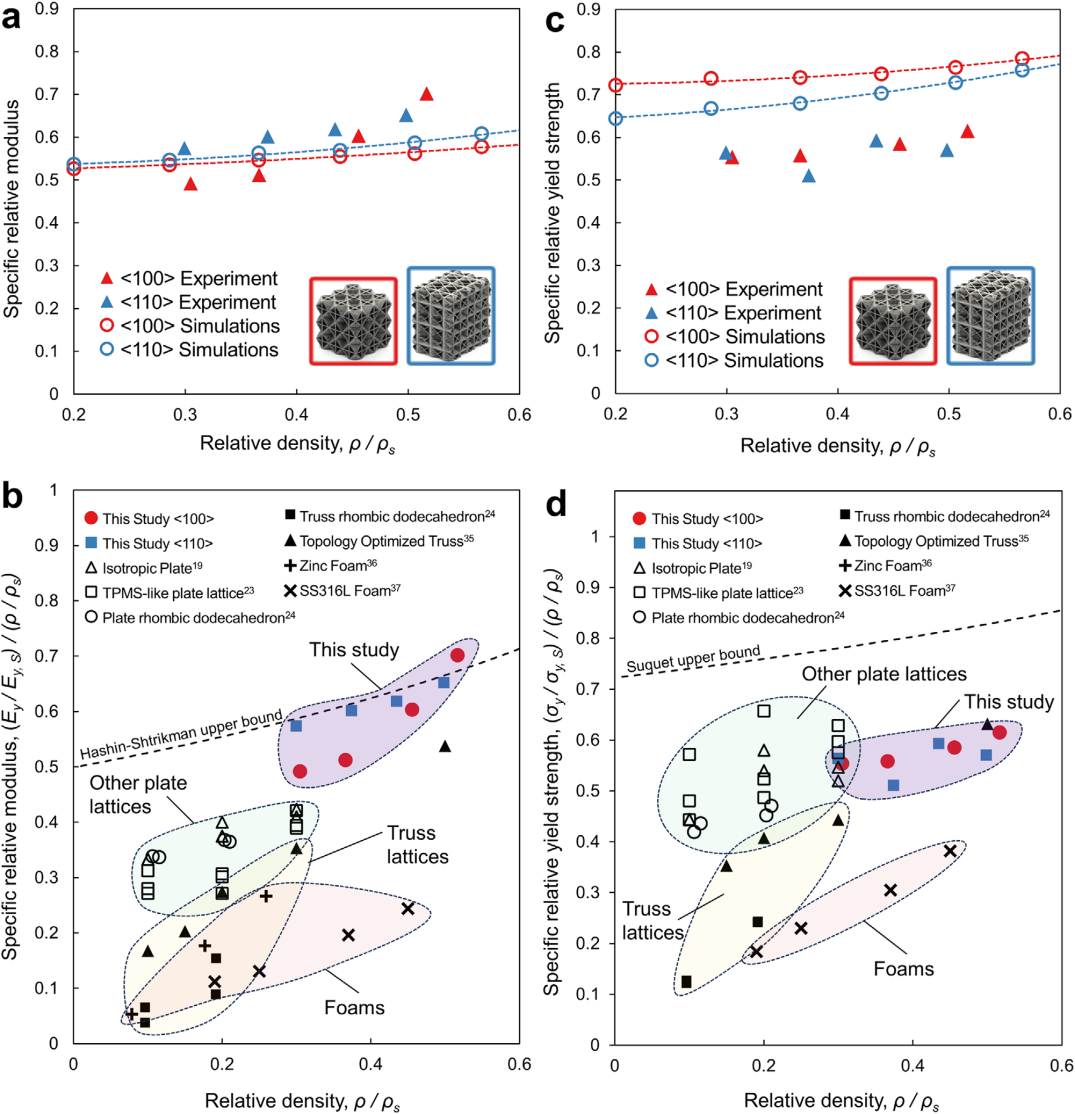
(a) Specific relative modulus ([*E/E_s_*]/[*ρ/ρ_s_*]) of <100> and <110> *pSC‐pFCC* closed cell microlattices, for *ρ/ρ_s_* = 0.29, 0.37, 0.44, and 0.51. (b) Comparison plots of specific relative modulus against relative density, with data points for other isotropic lattice designs and foams in the literature included for comparison [25, 29, 30, 41, 42, 43]. (c) Specific relative yield strength ([*σ_y_/σ_ys_*]/[*ρ/ρ_s_*]) of <100> and <110> *pSC‐pFCC* closed cell microlattices, for *ρ/ρ_s_* = 0.29, 0.37, 0.44, and 0.51. (d) Comparison plots of specific relative yield strength against relative density [25, 29, 30, 41, 42, 43]. For (a) and (c), solid data points represent experimental results while dotted lines with hollow data points represent simulation results. For (b) and (d), the black dotted lines represent the theoretical upper bounds.

Extracting the specific relative moduli (i.e. [*E/E_s_*]/[*ρ/ρ_s_*]) from the experimental stress‐strain curves and plotting them in Figure 6a, it can be observed that the values exhibited by the <100> lattices were very close to that of <110> microlattices—within 17% deviation—indicating that the *pSC‐pFCC* structures were elastically isotropic (Figure 6a). The experimental specific relative moduli were also in excellent agreement with the simulation results (Figure 6a), which followed the Hashin‐Shtrikman upper bound closely (Figure 6b). These observations are consistent with the predictions from previous analyses on the *pSC‐pFCC* geometry [15, 16] and presents a convincing validation of these theories, more so than previous studies because true closed cell structures faithful to the original *pSC‐pFCC* designs were used, as opposed to the pseudo‐closed cell lattices investigated to date [16, 25, 26, 27].

Compared to other isotropic porous materials, the metallic *pSC‐pFCC* closed cell microlattices in this study exhibited significantly better specific relative moduli (Figure 6b). This is consistent with previous studies, which showed that lattices and foams with truss‐like microstructures have a lower structural efficiency, and a correspondingly lower stiffness that can only reach up to ≈1/3 of the H‐S upper bound [15]. In addition, there has been significant difficulty in realizing the theoretical geometries of isotropic plate lattices using isotropic materials, which often caused the measured stiffness to fall short of the H‐S upper bound [25] (Figure 6b).

The isotropy in the elastic regime was also found to extend to the specific relative yield strength (i.e. [*σ_y_/σ_ys_*]/[*ρ/ρ_s_*]) of the microlattices – the values for the <100> and <110> microlattices deviated less than 10% from each other (Figure 6c). Unlike the specific relative modulus, however, the experimental values for the relative yield strengths were ≈20% lower than those predicted by simulations (Figure 6d), likely due to the presence of manufacturing imperfections that promoted premature buckling of the plates (Figure 3b). Nevertheless, the measured specific relative yield strengths remained comparable to other plate lattices reported in literature, and ≈2× as high as those typically observed in truss lattices and foams (Figure 6d).

#### 3.4.2 Energy Absorption Characteristics

Beyond stiffness and strength, the energy absorption of the metallic *pSC‐pFCC* closed cell microlattices were also characterized and assessed using the following indices of measure:

(a) Energy absorption efficiency, *η*, which can be computed using:

(2) $$\eta \;\left(\varepsilon \right)=\frac{1}{\sigma \left(\varepsilon \right)}\;\int_{0}^{\varepsilon }\sigma \left(\varepsilon \right)d\varepsilon =\frac{VEA}{\sigma \left(e\right)}$$

where *VEA* refers to the volumetric energy absorption, which is the energy required to mechanically deform a unit nominal volume of the lattice. For a given stress of *σ*, *η* is basically the actual volumetric energy absorbed (*VEA*), expressed as a fraction to the maximum volumetric energy that could theoretically be absorbed without raising the stress *i.e. σ* ∙ *ε*_max_. Since *ε*_max_ = 1 for compression, the denominator for Equation (3) is reduced to *σ*, which depends on the strain, *ε*. As a result, *η* is also a function of *ε*, and for stress–strain curves that undergo a stress plateau phase, the maximum *η* (*i.e. η*_max_) can be found at the densification strain, *ε*_d_, which marks the transition point between the stress plateau and densification regimes [44], i.e.

(3) $$\eta_{max}\;\left(\varepsilon \right)=\frac{1}{\sigma \left(\varepsilon_{d}\right)}\;\int_{0}^{\varepsilon_{d}}\sigma \left(\varepsilon \right)\;d\varepsilon =\frac{VEA}{\sigma \left(\varepsilon_{d}\right)}$$

At this point, the actual lattice exhibits peak performance by absorbing the most energy while transmitting the least stress. In other words, it behaves most like an ideal absorber when *ε* = *ε*_d_.

(b) Specific energy absorption (*SEA*), which can be expressed as:

(4) $$SEA=\frac{1}{\rho }\;\int_{0}^{\varepsilon_{d}}\sigma \left(\varepsilon \right)\;d\varepsilon$$

*SEA* indicates the energy absorbed per unit mass of the lattice. Like *VEA*, it varies with strain and hence, *SEA* is assessed at the point of maximum energy absorption efficiency, *η*_max_, which occurs when *ε = ε*_d_.

**FIGURE 7:**
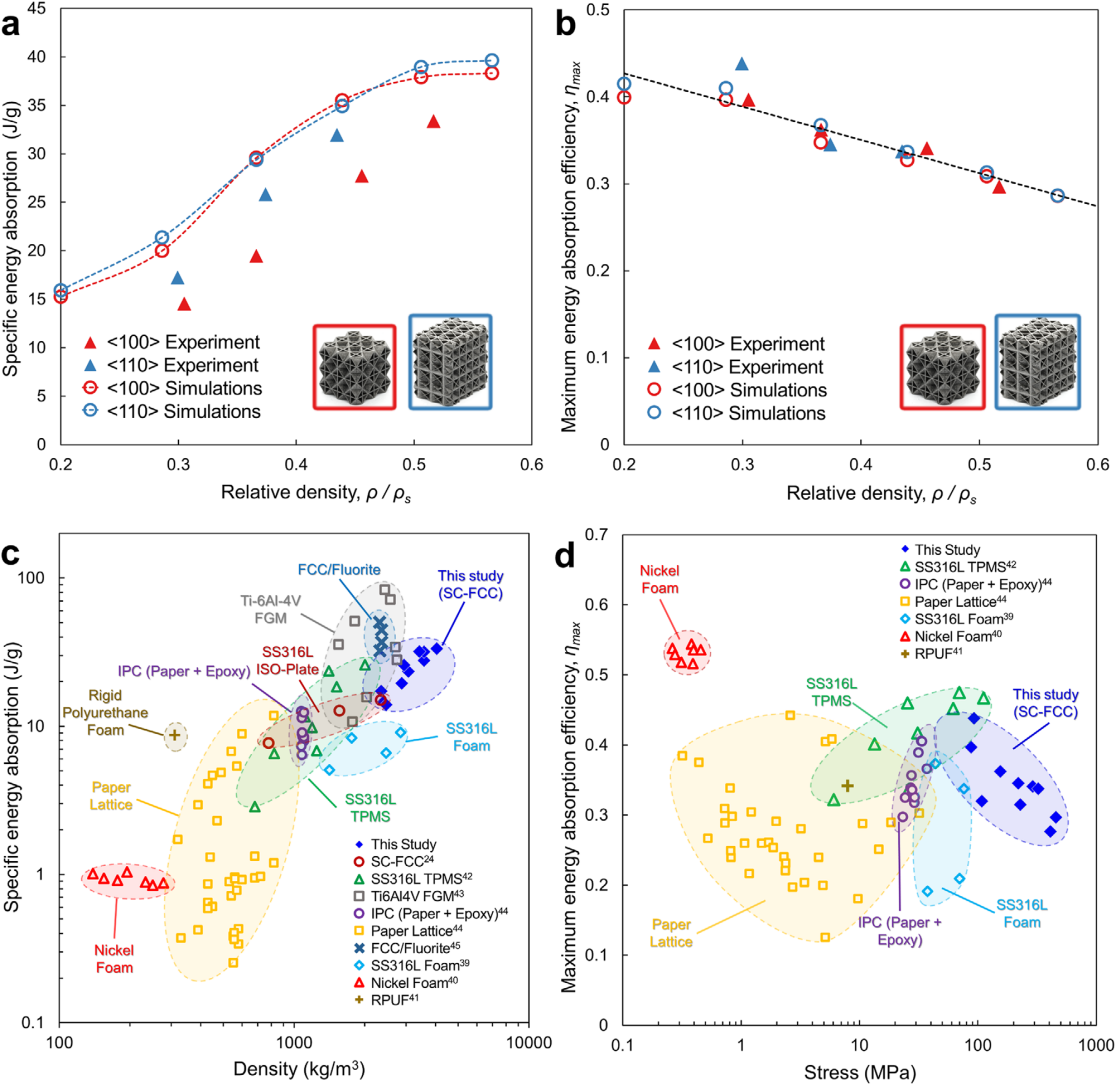
(a) Specific energy absorption (*SEA*) and (b) maximum energy absorption efficiency (*η_max_*) of <100> and <110> *pSC‐pFCC* closed cell microlattices, for *ρ/ρ_s_* = 0.29, 0.37, 0.44, and 0.51. (c) *SEA* vs density of the *pSC‐pFCC* closed cell microlattices in this study, compared to other foams [45, 46, 47], isotropic [30] and anisotropic lattices [18, 48, 49, 50] found in literature. (d) *η_max_* vs stress (at densification strain), compared to other foams and lattices [45, 46, 47, 48, 50].

From Figure 7a, it can be observed that FE simulations expected *SEA* of both the <100> and <110> orientations to be similar across all relative densities, which is expected since the simulated elastoplastic stress‐strain curves for both orientations were almost identical, as shown in Figure 5a. However, the experimental *SEA* values were consistently lower than the simulated values due to the reduced plateau stress of the actual lattices, which was in turn caused by imperfections in the vertical plates in the Build axis, as discussed earlier (Figure 3b). These imperfections may have introduced eccentricities that understandably affected the <100> microlattices, which failed by buckling, more than the <110> microlattices, which failed by diagonal shear, leading to a consistently lower *SEA* for <100> microlattices (Figure 7a). This discrepancy aside, the experimental trends for *SEA* vs. relative density actually agree very well with the simulations for the <100> and <110> microlattices. *SEA* has a positive correlation with relative density because an increase in material available for deformation leads to more energy absorbed.

Furthermore, the discrepancy between experimental and simulated *SEA* did not affect the results for *η*_max_, which showed an excellent agreement between the simulated and actual values (Figure 7b). This is because energy absorption efficiency is influenced more by the ‘shape’ of the stress‐stress curves, rather than the absolute stress values. Figure 7b also indicates that *η*_max_ for the <100> and <110> orientations are essentially indistinguishable, implying isotropy in energy absorption efficiency for the *pSC‐pFCC* microlattices. Unlike the case for *SEA*, however, *η*_max_ can be observed to decrease linearly with increasing relative density. This can be explained by the increased void fraction at low relative densities, which allows the plates in the microlattice to deform more extensively before coming into contact with each other. This leads to a long stress plateau regime and high densification strains, which will mathematically produce larger energy absorption efficiencies.

Benchmarking the *SEA* of the SS304L *pSC‐pFCC* microlattices against a range of other lattices and foams (Figure 7c), it can be observed that the *SEA* of the *pSC‐pFCC* microlattices is one of the highest in the literature at 14 – 33 J g^−1^, outperforming TPMS lattices and isotropic stainless steel foams with comparable densities. In fact, the *SEA* of the isotropic *pSC‐pFCC* microlattices were so high that they were within the range of *SEA* for anisotropic Ti6Al4V functionally graded lattices, which had been optimized to absorb energy only in specific directions [49].

Going further, we also note that the energy absorption efficiencies of the SS304L *pSC‐pFCC* closed cell microlattices are comparable to or even exceed those of other reported metamaterials, reaching a value as high as 44% (Figure 7d). Typically, such high energy absorption efficiencies can only be achieved at relatively low stresses, with constituent materials that are highly porous [46] and/ or polymeric [50] in nature. However, the excellent structural integrity of the *pSC‐pFCC* geometry, together with the work hardening of the constituent SS304L material, enabled the metallic microlattices to attain these efficiencies without compromising the strength of the metamaterials. As a result, *η*_max_ = 0.27 to 0.44 was demonstrated at stresses up to 410 MPa (Figure 7d), indicating that these microlattices can be useful even under extreme loads.

## 4 Discussion

In recent years, there has been considerable research directed toward the design of isotropic plate lattices. Manufacturing limitations, however, mandate the addition of holes to the geometries to facilitate precursor material removal [16, 17, 25, 26, 27]. This compromises the structural properties of the metamaterials, leading to at least 5% reduction in stiffness [27] and 13% – 16% decrease in yield strength when the hole diameter exceeds 10% of the unit cell length (Figure S6). Even with these release holes, however, remnants of the precursors often remain trapped within the innermost parts of the pseudo‐closed cell structure, particularly in large lattices with many unit cells [17]. This implies that the technique of introducing release holes has limited scalability for practical engineering parts. Moreover, when the mass of the trapped material is not accounted for because lattice properties are normalized against theoretical relative densities [16, 26, 27], the specific relative modulus and specific relative strength of the lattices may become erroneously inflated.

Other than the trapping of precursors, material isotropy has also remained a largely unverified assumption in previous works. AM methods such as PBF [51] and FDM [52] are known to introduce material anisotropy through the energy/ material deposition paths, as well as the layer‐by‐layer fabrication sequence which produces geometrical deviations such as the staircase effect [53]. Post‐print heat treatments often offer only modest improvements in isotropy, with the prints remaining largely anisotropic [54]. Despite these limitations, prior studies on pseudo‐closed cell lattices have often assumed that the structures exhibit identical mechanical response along both the Build and Transverse axis without experimental verification [16, 17, 25, 26, 27]. In studies where 2 photon polymerization was used to fabricate microscopic lattices, for instance, the extreme size of these structures makes it virtually impossible to test the material in any direction other than the Build axis [16, 27].

To circumvent these limitations, the present LAPIS technique removes excess material at every layer, enabling complete precursor material evacuation without the need to introduce holes. The self‐supporting precursor sheets allow new layers to be added over voids in preceding layers, which removes the need for support structures within the cavities and enables complete sealing of the enclosed spaces in the lattice. Micro‐CT scans confirmed that the enclosed cavities were indeed vacant, with experimental relative densities deviating less than 4% from the design values. Furthermore, post‐print heat treatment was carried out to restore isotropy to the constituent material after printing, which was experimentally verified through testing in the Build and Transverse axis. The successful realization of the *pSC‐pFCC* design not only enabled us to validate predictions of its exceptional elastic stiffness, but also provided new insights into its plastic response. Although simulations in this study (Figure 5a; Figure S5), as well as previous publications [55], can be utilized to investigate and understand the large‐scale deformation of plate lattices, the present work is the first time these findings have been experimentally verified. Among the most significant insights is the fact that *pSC‐pFCC* metallic microlattices exhibit significant isotropy in the plastic regime as well, despite failing via different mechanisms in different orientations (Figure 5a; Figure S5).

It has traditionally been assumed that stretch‐ or compression‐ dominated lattices, despite demonstrating high relative modulus, are poor candidates for cushioning applications [56]. While their rigidity allows high peak stresses to be sustained in the elastic region, failure in the lattice elements, which usually proceeds via softer bending or buckling deformation, tends to cause a sudden drop in load‐bearing capacity. This effect is particularly pronounced in cubic lattice designs, where failure occurs layer‐by‐layer, producing oscillations in stress throughout the compression process that could damage payloads due to the sudden acceleration and deceleration [39, 57, 58]. In contrast, bending‐ or rotation‐dominated lattices undergo the same deformation mode in both elastic and plastic regimes, facilitating a smoother stress‐strain transition, but their soft deformation mechanisms preclude a high elastic stiffness and strength [59, 60, 61].

However, the present work demonstrates that material work hardening, often neglected in the general analyses of lattice structures [26, 30, 62], can enable a lattice to combine high rigidity with excellent energy absorption, allowing the best characteristics of stretch‐dominated and bending‐dominated designs to be united within a single metamaterial. This is because work hardening can strengthen the constituent material after lattice failure, leading to high stresses sustained by the lattice even as it undergoes ‘soft’ buckling or shearing deformation modes. These stresses would be comparable to the yield stress, which was attained with a weaker, pre‐work‐hardened material undergoing ‘stiff’ compression/ stretching deformation in the elastic regime. The net effect of this is a smoothing of the stress‐strain response of the metallic microlattices from the elastic to plastic regime, which led to energy absorption efficiencies comparable to those of plastic foams, but at stresses and volumetric energy absorption, two orders of magnitude higher.

Taken together, these findings demonstrate that the metallic *pSC‐pFCC* closed cell microlattice is uniquely positioned for high‐performance, multi‐axial loading applications. Its isotropy, coupled with high stiffness, strength, and excellent energy absorption capabilities, makes them ideal for demanding applications such as acetabular cup implants [63], where long‐term structural reliability under multidirectional physiological loading is essential, as well as in aerospace and automotives, where crashworthiness demands excellent energy absorption efficiencies at stresses in the range of 100 – 300 MPa [64, 65, 66]. These benefits can potentially be realized through the LAPIS technique presented in this work, which offers a practical pathway for translating and scaling these closed cell microlattice designs into real world engineering applications.

## 5 Conclusions

*pSC‐pFCC* closed cell microlattices were successfully fabricated from SS304L sheets via the LAPIS technique, without the introduction of release holes for precursor material removal – a common compromise in earlier studies using alternative AM methods. To restore material isotropy, a post‐print heat treatment was employed to promote grain recrystallization and microstructural uniformity. Compression tests conducted for the <100> and <110> orientations across varying relative densities confirmed that the microlattices exhibited significant isotropy in the elastic deformation regime, with stiffnesses close to the Hashin‐Shtrikman upper bound, consistent with previous predictions. This isotropy was extended to the plastic deformation regimes as well, despite the microlattices failing by different mechanisms in different orientations. The experimental yield stress and plateau stress of the microlattices in the Build axis were slightly lower than that expected from simulations, due to manufacturing imperfections. In addition, work hardening of the metallic material was found to bridge the high stresses sustained by the microlattices in the elastic regime with the buckling or shearing failure in the plastic regime, leading to remarkable specific energy absorption (*SEA*) values of 15 – 33 J g^−1^ and energy absorption efficiencies up to 44%, at stresses up to 410 MPa.

## Author Contributions

**Dominic Kang Jueh Lim**: Conceptualization, Methodology, Validation, Investigation, Formal analysis, Writing – Original Draft. **Lai Changquan**: Methodology, Formal analysis, Supervision, Funding acquisition, Conceptualization, Writing – review & editing, Resources.

## Conflicts of Interest

The authors declare no conflicts of interest.

## Supporting information




**Supporting File**: smll73694‐sup‐0001‐SuppMat.docx.

## Data Availability

The data that support the findings of this study are available from the corresponding author upon reasonable request.
