# Supplementary material for: Ultrahigh Stiffness and Energy Absorption Properties of Isotropic Metallic Closed Cell Microlattices
Source: Small. 2026 May 7;22(35):e73694. doi: 10.1002/smll.73694 (PMC13288809; doi:10.1002/smll.73694)
Supplement: Supplementary file 1 — Supporting File: smll73694‐sup‐0001‐SuppMat.docx. [file SMLL-22-e73694-s001.docx]

**Supplementary Information**

**Ultrahigh Stiffness and Energy Absorption Properties of Isotropic Metallic Closed Cell Microlattices**

Dominic Kang Jueh Lim^a^ & Chang Quan Lai^a,b,c,*^

*^a^ School of Mechanical & Aerospace Engineering, Nanyang Technological University, 50 Nanyang Ave, Singapore 639798, Singapore*

*^b^ Singapore Centre for 3D Printing, School of Mechanical & Aerospace Engineering, Nanyang Technological University 639798, Singapore*

*^c^ School of Materials Science & Engineering, Nanyang Technological University, 50 Nanyang Ave, Singapore 639798, Singapore*

**Keywords:** Additive manufacturing; microlattice; metamaterials; plate lattice; closed cell; energy absorption

*Corresponding author: [cqlai@ntu.edu.sg](mailto:cqlai@ntu.edu.sg)


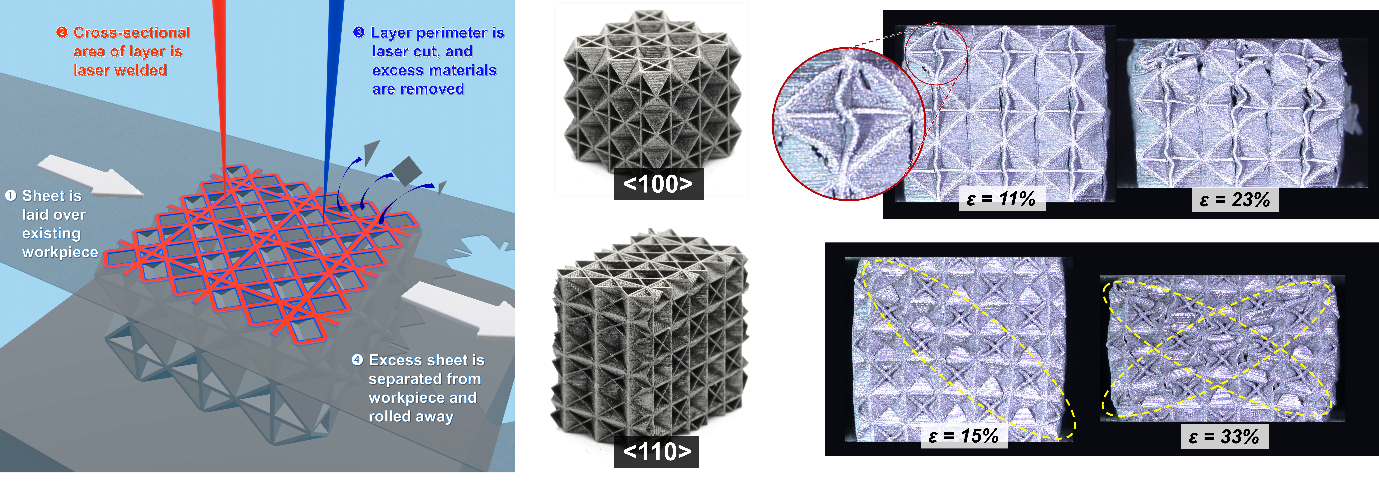


**
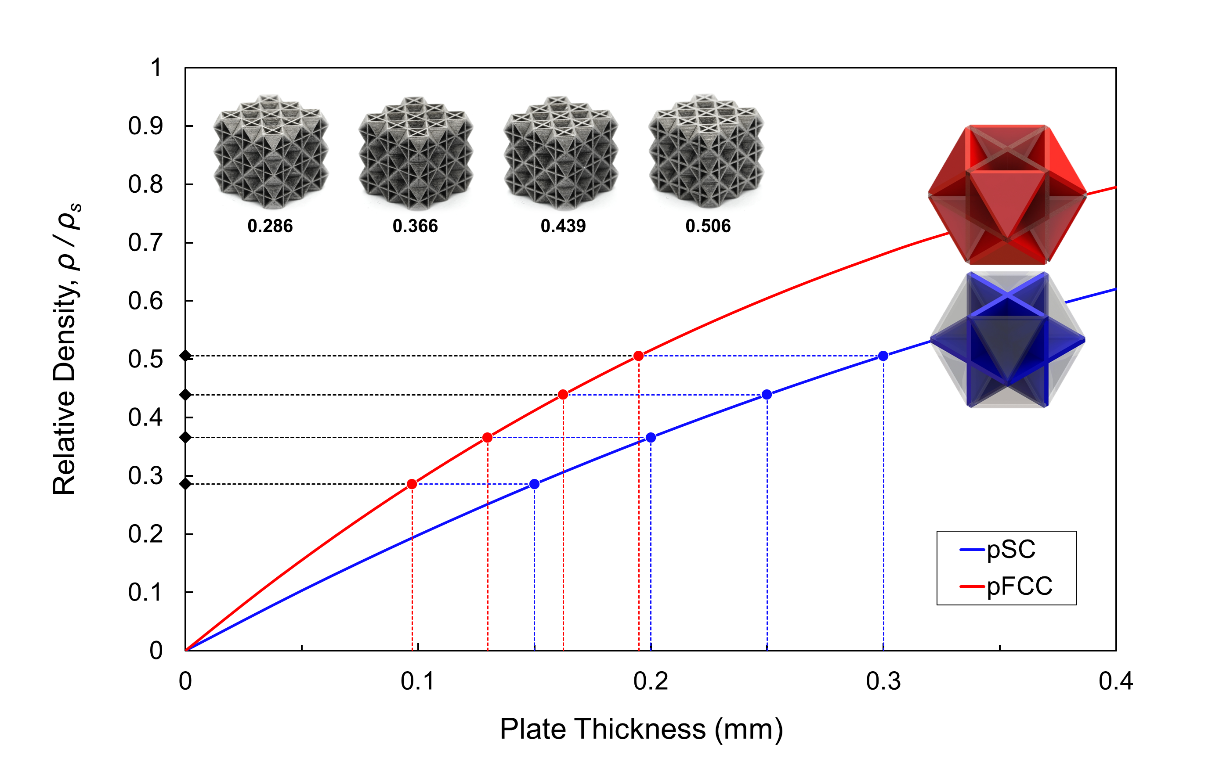
**

**Supplementary Fig. S1** | Relative density of *pSC-*pFCC microlattices and corresponding plate thicknesses of *pSC* and *pFCC*.


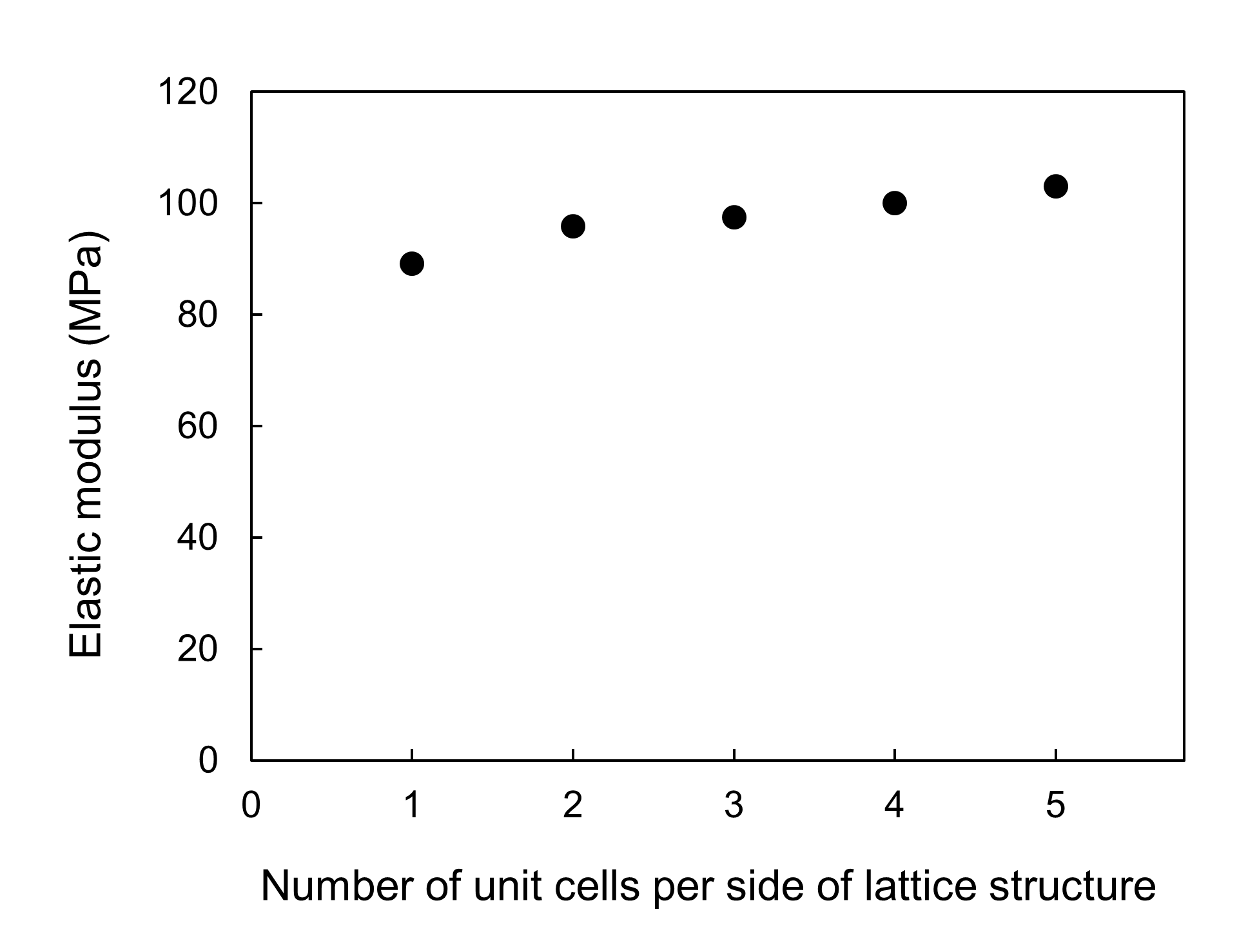


**Supplementary Fig. S2** | Elastic modulus vs number of units cells used per side of the *pSC-pFCC* microlattice. Size effect was simulated using <100> microlattice with *ρ/ρ_s_* = 0.29.


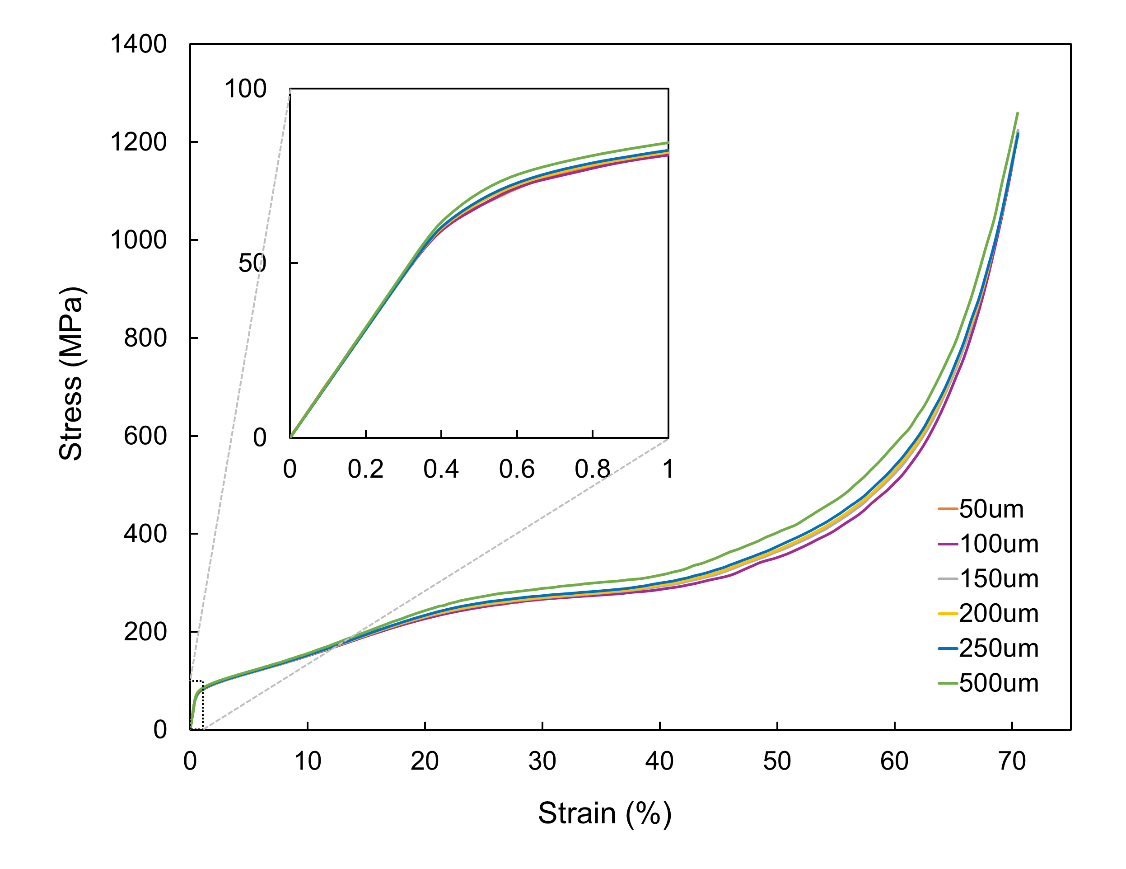


**Supplementary Fig. S3** | Mesh convergence on a *pSC-pFCC* <100> microlattice with *ρ/ρ_s_* = 0.44, depicting the simulated stress-strain curves for mesh size of 50μm to 500μm. Results show good convergence when mesh sizes fall below ~ 250μm.


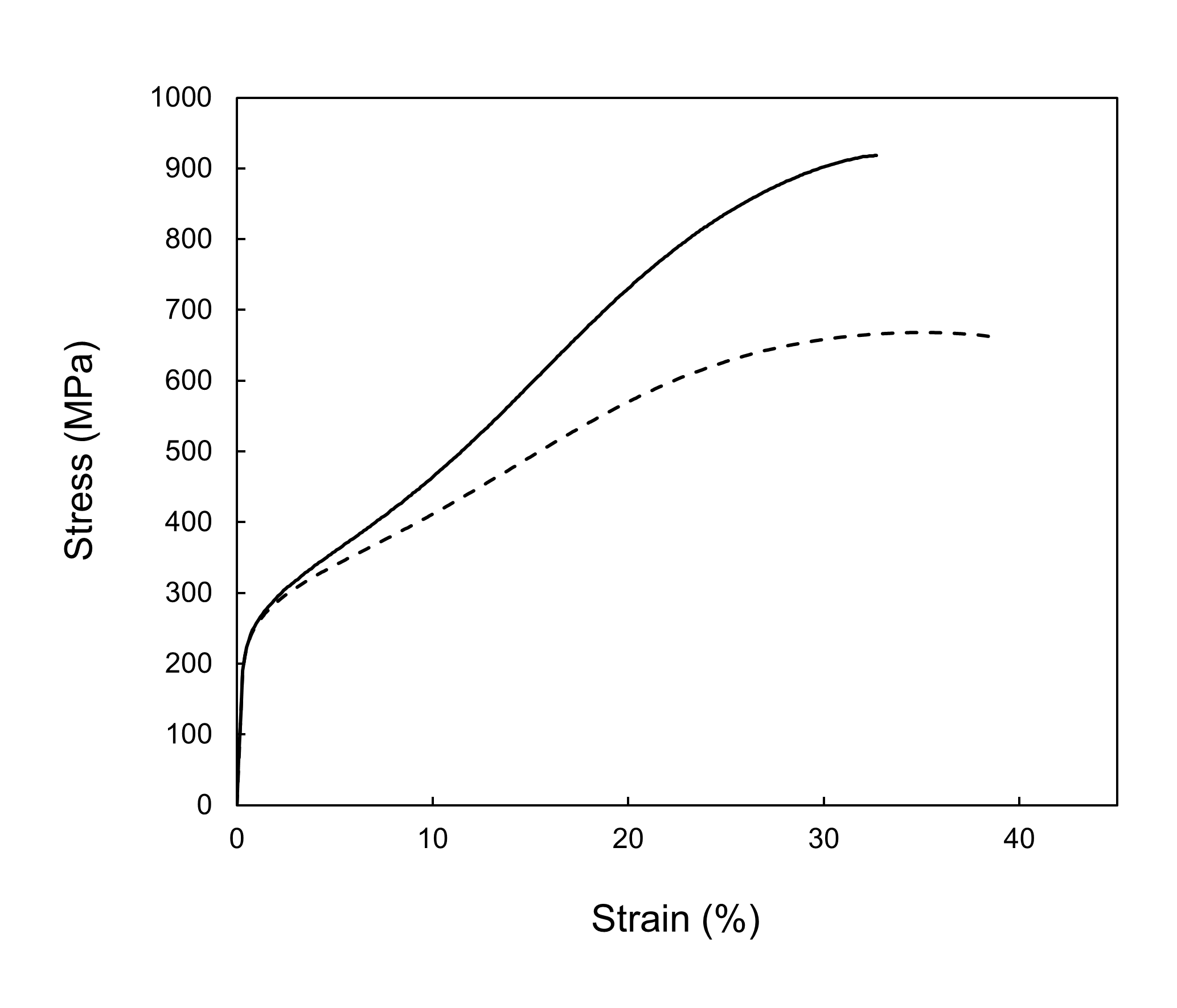


**Supplementary Fig. S4** | True stress-strain data used in the finite element simulations, obtained from uniaxial tensile test of an annealed SS304L tensile specimen.


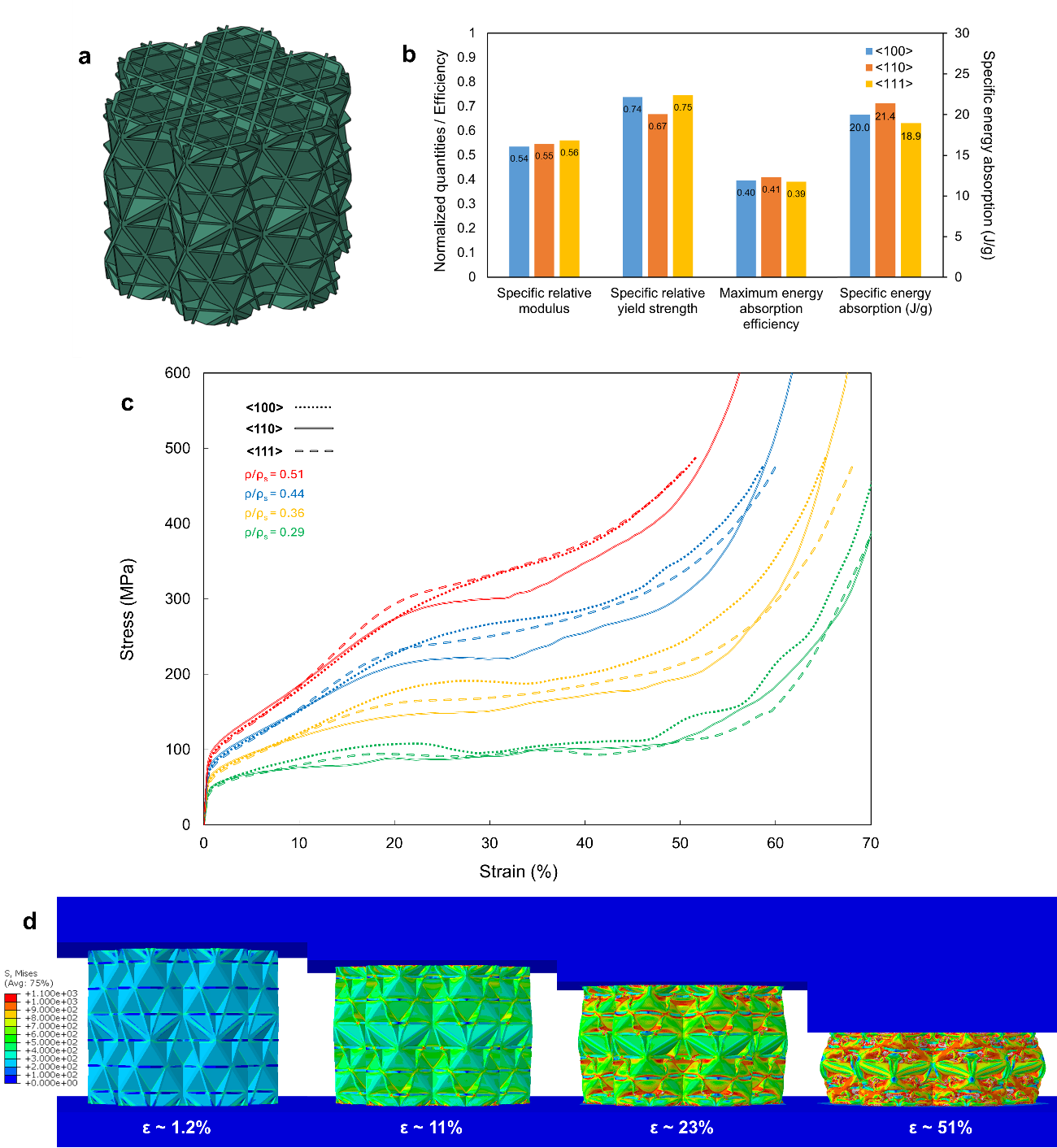


**Supplementary Fig. S5** | (a) *pSC-pFCC* microlattice model for compression along <111> axis. A hexagonal geometry was adopted to ensure tessellation and eliminate edge effects. (b) Comparison of elastic and plastic mechanical properties along <100>, <110> and <111> orientations for a *pSC-*pFCC microlattice with relative density of 0.29. (c) Simulated stress-strain response of a *pSC-pFCC* microlattice under compression along <100>, <110> and <111> orientations across different relative densities. (d) Simulated uniaxial compressive response of <111> oriented microlattice.

The additional simulations in Figure S5, conducted along the <111> orientation, further confirm that the yield strength, specific energy absorption, and energy absorption efficiency are comparable across orientations, particularly at lower relative densities *e.g.* for a relative density of 0.29, the differences among the <100>, <110>, and <111> orientations were within 5%, demonstrating a high degree of plastic isotropy.


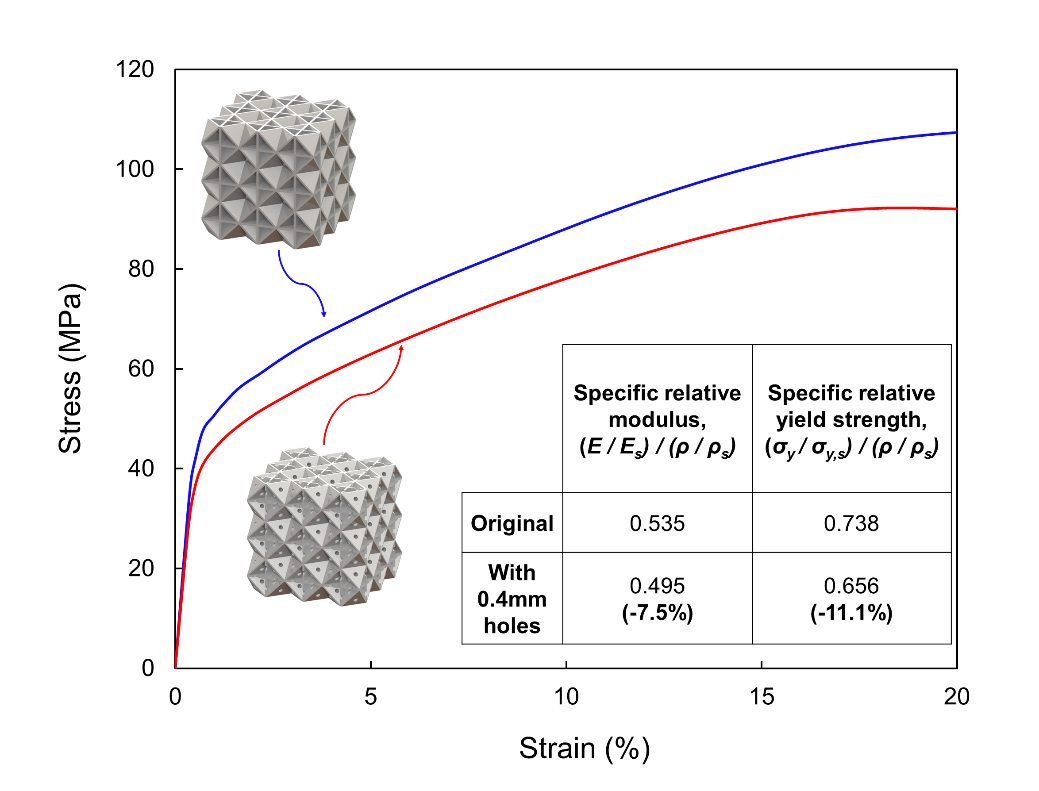


**Supplementary Fig. S6** | Simulated stress-strain response of a *pSC-pFCC* <100> microlattice with and without the incorporation of 0.4mm diameter holes.
